# Intraspecific interactions in spring-staging geese reflect mate guarding and proximity to nesting dates

**DOI:** 10.1038/s41598-026-43082-x

**Published:** 2026-03-16

**Authors:** Michał Polakowski, Łukasz Jankowiak, Anthony David Fox

**Affiliations:** 1 Department of Ecology and Anthropology, Institute of Biology, University of Szczecin, Wąska 13, Szczecin, 71-412 Poland; 2 Department of Ecoscience, Aarhus University, C.F. Møllers Allé 8, Aarhus C, 8000 Denmark

**Keywords:** Wild geese, Intraspecific aggression, Flock size, Mate-guarding, Spring stopover, Breeding timing, Ecology, Ecology, Zoology

## Abstract

Wild geese form large flocks, benefiting from shared vigilance against predators which increases feeding time. As spring progresses, males invest more in mate-guarding to protect paternity, so we hypothesised that intraspecific aggression would rise with flock size and proximity to first egg dates. We tested this using field observations of four goose species staging in northeast Poland, February–April: one local breeder (nesting in March) and three Arctic-nesting species (laying from late May). Of 662 observed aggressive episodes, 643 (97%) were intraspecific, and aggression in all species increased with flock size. Locally breeding Greylag Geese *Anser anser* and Arctic-nesting Barnacle Geese *Branta leucopsis* showed consistently high probabilities of aggression (60–80%) throughout, although Barnacle sample sizes were too small for firm conclusions. The other two Arctic-nesting species (Greater White-fronted *Anser albifrons* and Tundra Bean Goose *A. serrirostris*) showed aggression rising initially from zero to similarly high levels in the three weeks before departure for nesting areas. No differences were found between geese feeding on grassland versus arable fields (which offered higher food intake). We conclude that the predominance of intraspecific aggression is consistent with increasing mate-guarding (and related close-range social defence) as spring progresses relative to species-specific nesting schedules, and is unlikely to be driven primarily by interspecific competition for food or other types of interactions.

**Supplementary Information:**

The online version contains supplementary material available at 10.1038/s41598-026-43082-x.

## Introduction

Wild geese in the non-breeding season are classic flocking species, benefitting from shared vigilance and enhanced feeding benefits of dense feeding aggregations, since individual time spent alert decreases with flock size^1–3^. Intra-specific aggression occurs, mostly related to the dominance of family units of greatest size over other groups and individuals, enabling parents to secure best feeding opportunities for their offspring during their first winter, although ganders of newly formed pairs can win agonistic interactions with larger families^4,5^. Family break-up often occurs through winter until arrival to the breeding areas depending on individuals and species^6^, although parent-offspring and sibling-sibling can persist for longer in some goose species and up to 13 years in some populations of Greater White-fronted Geese *Anser albifrons*^7–10^. Up to 60% of Barnacle Geese *Branta leucopsis* goslings are unattached by spring departure from the wintering grounds, due to increasing parent-offspring aggression, but parents associating with offspring until departure were more experienced, showed higher survival and were more likely to breed successfully than those without their young^11^. As the spring advances, therefore, the imperative of a paired gander evidently shifts increasingly from being a flock member, enjoying shared vigilance and enhanced feeding time for himself, his mate and offspring to ensure survival, to investing in the ability of his female to attain the best possible body condition for investment in forthcoming reproduction. In spring, paired adult males are the most aggressive of geese and the determinant of rank within flocks^5^. They must trade-off the advantages of tolerating near associates (kin, including the young of the last summer and previous years while the benefit of their protection outweighs the burden^11^ and unrelated geese (benefitting from reduced vigilance and increased food intake rate) versus the need to protect his female to maximise her body condition for investment in successful reproduction in the coming breeding season. Attendant ganders must also increasingly secure their mating rights over other males to ensure their paternity in ultimate reproductive attempts. Hence, we also expect mate guarding to increase towards the time of first egg dates^12^, given the prevalence of forced extra-pair copulations and the potential for sperm storage in these species^13,14^. As the spring progresses, the net above ground productivity of graminoids increases, relaxing the food limitations imposed on foraging geese by short daylengths and reduced plant growth, potentially reducing the benefit of flocking because of the costs of competition. During spring migration, migrating geese often aggregate into even larger flocks, when increased group-size may result in intensified competition for food and/or conflicts between monogamous ganders over mates resulting in enhanced aggression^15,16^, both of which may are likely to be enhanced during migration and pre-breeding. Reproductively active pairs within large goose flocks need to increasingly invest in the accumulation of necessary fat loads and nutrient store for their onwards migration and for the female’s investment in a clutch of eggs and her self-maintenance through incubation^17–19^. As a result, geese, like other spring migrant birds, aggregate at sites which sustain high food intakes rates^19–21^, often at high feeding densities. Here, they face increasing conflicts between the benefits of being a flock member and the increasing urgency of their coming investment in reproduction on arrival to breeding areas. Since temperate nesting geese breed earlier than those nesting in the low and high Arctic, these transitions likely occur at different points in the annual calendar resulting in asynchrony between different goose populations occurring in large mixed flocks on spring staging areas.

For all these reasons, in flocks of spring staging geese of mixed species, we hypothesise an increase in inter- and intra-specific aggressive interactions as spring migration progresses, because of the effects of competition for limited food resources (both inter- and intraspecific, but site specific and not necessarily time dependent) and/or increased mate guarding (hence, exclusively intraspecific, not site specific and dependent on closeness to the first egg dates of different species). Here, for the first time, we test these predictions by analysing spring staging goose behaviour patterns among four species occurring together at an important staging area in Central Europe, the Biebrza Basin in Poland. Staging flocks predominantly comprised three species staging en route to breeding areas in the Russian Arctic: the Tundra Bean *Anser serrirostris*, Greater White-fronted and Barnacle Goose, as well as the locally breeding Greylag Goose *Anser anser*, all of which have shown recent local and population increases in abundance^22,23^.

Based on the predictions above, in response to increasing food competition, we predict that (i) as flock sizes increase, both inter- and intra-specific aggressive encounters would increase with date across all species and would not be time dependent. We might also predict (ii) that given that geese maintain higher food intake rates on cereal fields compared to grassland^24^ aggression would be more frequent on cereal than grass. Furthermore, if mate guarding was playing an increasing role in initiating aggressive encounters between geese, we would expect to see (iii) increasing agonistic but purely intra-specific encounters through the spring staging period which may or may not be related to flock size. We would also expect this process to start earlier and be more evident among the locally breeding species (i.e. Greylag Geese that are closer to their ultimate first egg dates and are partially local breeders) than the Arctic nesting species (i.e. Barnacle, Tundra Bean and Greater White-fronted Geese that will continue staging across continental Europe onwards to their ultimate Arctic breeding areas).

## Methods

### Study area

This study was conducted in the Biebrza Basin, northeastern Poland. This area is recognized as an Important Bird Area for both breeding and migratory bird species^25^ and serves as a key migratory stopover in Central Europe for numerous waterbirds^26^, due to the presence of extensive seasonal floodplains associated with two river systems—the Biebrza (100 km in length) and the Narew (65 km in length). The Biebrza Basin is one of the principal spring staging areas in Central Europe for Greater White-fronted Geese en route to their Siberian breeding grounds, and is an important stopover for Barnacle Goose, Tundra Bean Goose, and Greylag Goose, all of which have been the subject of ornithological studies^3,22,24,27-30^. The Biebrza Basin extends to *c.* 54,326 ha, comprising 13,689 ha of arable lands (mostly winter cereals), 28,260 ha of grasslands (grazed or mown meadows and pastures, typically at lower elevations subject to seasonal flooding) and wetlands^22,30,31^. Geese form mixed-species flocks of up to 40,000 individuals in this area, with an increasing proportion currently foraging on arable fields, in contrast to the previously preferred use of grasslands^3,22,24,27^.

### Field studies

Observations were conducted on 26 days between 9 February and 10 April 2024 (totalling 222 h) throughout the entire goose spring staging period in the Biebrza Basin^27^. Staging goose numbers rarely exceeded 1,000 individuals until mid-March, with numbers peaking between 19 and 22 March, when the Greater White-fronted Goose was overwhelmingly dominant and numbers exceeded 80,000 individuals. Goose numbers declined rapidly after 25 March, when flock sizes did not exceed 1,700 individuals. Observations were carried out throughout the Biebrza Basin, but more in the south, namely Wizna Marsh and the Southern Biebrza Basin, where goose concentrations are typically highest^27^. For each flock observed, we determined species composition and the number of individuals per species before the detailed behavioural observations took place.

We also identified foraging habitat types used by the birds, distinguishing between arable fields (mainly winter cereals and stubbles) and grasslands (meadows and pastures). A total of 29,353 geese were recorded foraging in arable fields, with 90.8% grazing on winter cereals compared with 131,207 individuals observed feeding on grasslands grounds.

A Swarovski ATS 80HD spotting scope (25–50x zoom) was used to count and identify species within flocks, while Swarovski NL Pure 8 × 32 binoculars were used interchangeably depending on the distance from the birds to detect behavioural interactions. To minimize disturbance, observations were conducted from distances of up to several hundred meters.

### Behavioural observations

We employed scan sampling [*sensu*^32^] to gather instantaneous data on the frequency of aggression between geese of all species. We did so because the relative rarity of such behaviours renders this technique more effective than deriving data on individual birds followed during prolonged focal sampling. Throughout the study, one observer continuously scanned back and forth through the flock using wide-field binoculars, allowing for detection and recording of aggressive behaviours (in contrast to all other non-aggressive behaviours e.g. foraging or resting, which were not recorded). We quantified aggression using standardized five-minute observation bouts of foraging goose flocks (*n* = 743 flock-level bouts). For each separate flock observation bout, we recorded: (i) species composition and (ii) abundance (species-specific count abundance denotes the number of individuals of the focal species present in the flock at the start of the five-minute bout), determined before behavioural recording using a spotting scope; (iii) the number and nature of aggressive behaviours, recorded as any clear antagonistic interaction within or between species. Within each five-minute bout, we continuously scanned through the flock and recorded the frequency of aggressive encounters observed among flock members. For each aggressive encounter we noted the species of the aggressor and, the recipient species, and subsequently categorized encounters as directed toward conspecifics or toward individuals of other species.

We defined aggression in geese as the following behaviours: chasing, pursuing, hissing, pecking attempts, and lowered head/neck posturing indicative of readiness to attack. The first and the last of these behaviours were those which were most typical of mate-guarding in all species. An encounter was defined as a single occurrence of any of these behaviours. Each case of aggression was classified as a separate event when it was clearly separated from the preceding one by a period of non-aggressive behaviour, such as foraging or resting. Such behaviour usually involved single individuals (i.e., one bird attacking another), whereas only occasionally did it occur as aggressive interactions involving several individuals simultaneously (*n* = 9). In one case, three separate aggressive events by the same individual directed toward different conspecifics were recorded as discrete aggressive events.

Importantly, because very large flocks make it difficult to track and identify participants reliably, we made no attempt to count how many individuals “participated” in each encounter. However, given the monogamous nature of pair bonds in geese and the need to prepare the female for energetically costly reproduction, we expect that the primary, and often sole, participant in conflicts with other geese is the male guarding his partner. Instead, our behavioural data represent the frequency and nature of aggressive encounters detected during standardized five-minute observation bouts (including the species identity of the aggressor and recipient).

Because the four goose species differed in absolute and relative abundance and in the number of bouts in which they were present, we reported both absolute counts of aggressive encounters and standardized frequencies. For each species, we calculated (i) the absolute number of aggressive encounters initiated by that species (event counts), and (ii) a standardized frequency expressed as events per 1,000 individuals scanned (total events initiated by a species divided by the summed abundance of that species across all bouts, ×1000).

### Statistical analysis

#### Model fitting

All statistical analyses were conducted in R (version 4.1.3; R Core Team). We fitted binomial generalized linear mixed-effects models (GLMMs) with logit link using the package lme4.

#### Response variable and sampling unit

Our analyses used the five-minute observation bout as the basic statistical sampling unit. For each bout and for each species present during that bout, we recorded whether that species initiated any aggressive behaviour. This produced a binary outcome at the species × bout level: either observed (coded as 1) or not observed (coded 0). Within the binomial GLMM, this binary response was entered in the standard 1/0 format used by lme4, using a two column response of the form cbind(yes_agress, non_agress), where yes_agress is the indicator for aggression (1 or 0) and non_agress = 1 − yes_agress. Each row in this dataset corresponds to a single species x bout combination and therefore to a single Bernoulli trial: a yes/no outcome describing whether aggression by a given species occurred during that particular five-minute observation bout. This formulation means the model estimates the probability that a given species showed aggression during an observation bout, rather than the number of birds “involved” in aggression versus those that were not within each bout.

#### Predictors and data preparation

For each bout we recorded date and time, which were converted for analysis to day-of-year and continuous hour (decimal hour), respectively. We also included habitat (grassland or winter cereal field). Because the chance of observing at least one aggressive act increases with the number of potential individuals, we included the log-transformed number of individuals of the focal species present in the flock (log(abundance)) as a covariate.

To account for non-independence due to repeated observations within the same flock, we included a random intercept for flock (flock_id). The numeric flock_id was assigned to group observation bouts from the same flock (date × location, and continuity of flock composition); when flock composition clearly changed (e.g., addition of different species), a new identifier was assigned.

Potential predictor variables were checked for multicollinearity using variance inflation factors (VIF), ensuring values remained below commonly used thresholds (VIF < 3).

#### Model selection

We began by specifying the model with most parameters that included the main effects of species, day of year, hour of day, and log(abundance), as well as selecting a priori two-way interactions considered biologically interpretable: species × day of year, species × hour of day, and species × log(abundance). These interactions test whether species differ in seasonal changes in aggression, diel changes in aggression, and density/opportunity-related changes in aggression, respectively. We did not pursue higher-order interactions because they are less interpretable and were not supported reliably by the data structure. To account for non-independence arising from multiple observation bouts originating from the same site-day flock context, we included a random intercept for flock_id.

We evaluated interaction terms using backward stepwise model simplification: we sequentially dropped interactions from the most parameterized model and compared nested models using likelihood-ratio tests. Interactions that did not improve model fit (*p* > 0.05) were removed step by step. After determining the supported interaction structure, we continued removing non-significant main effects or remaining terms in a stepwise way, again guided by likelihood-ratio tests, retaining terms with *p* < 0.05.

#### Habitat-specific analyses

To assess whether habitat altered aggression patterns, we repeated the above modelling procedures for bouts observed in grasslands and winter cereal fields (*n* = 701), using the same GLMM framework (binomial error, random intercept for flock_id, the same candidate interactions among species, day of year, hour, and log(abundance)). Likelihood-ratio tests again guided term removal.

## Results

### Relative frequency of intra- and inter-specific aggressive interactions

**Fig. 1:**
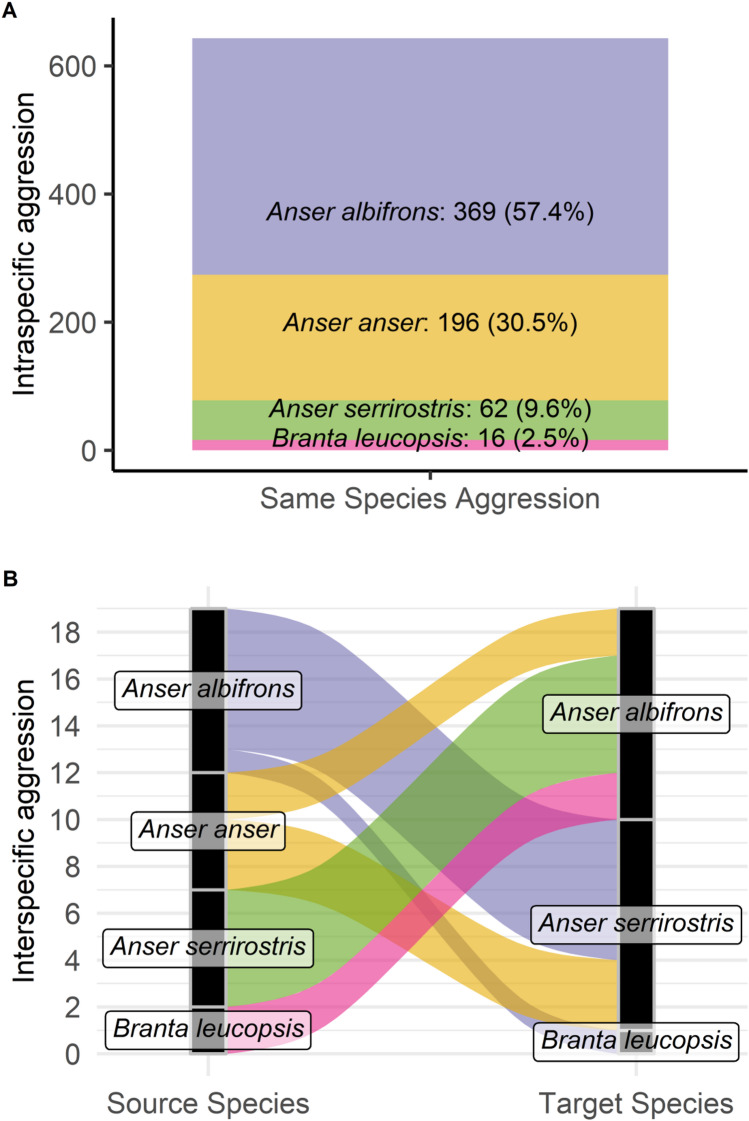
Total number of aggressive interactions observed among four staging goose species (Great White-fronted *Anser albifrons*, Greylag Goose *Anser anser*, Tundra Bean *Anser serrirostris*, Barnacle Goose *Branta leucopsis*) in the Biebrza Basin during spring 2024. Observations are divided between intra-specific (**A**, stacked histogram) and inter-specific (**B**, Sankey diagram) showing source and target species involved in the observations.

Intra-specific aggression (643 observations, constituting 97% of 662 aggressive cases), far exceeded the number of observations of inter-specific aggression (19 observations) throughout the entire spring staging period. In absolute terms, Greater White-fronted Geese (369 cases, 58%) and Greylag Geese (196 cases, 31%) showed the greatest levels of intra-specific aggression, with Tundra Bean Geese (62 cases, 10%) and Barnacle Geese (16 cases, 3%) contributing considerably less (Fig. 1A). However, when standardized per 1,000 individuals, Greylag Geese showed by far the highest relative frequency of intra-specific aggression (29.3 events / 1000 ind.), followed by Barnacle Geese (9.0), Tundra Bean Geese (4.0). The numerically dominant Greater White-fronted Geese showed least aggression of all (2.7). During the staging period, the total cumulative number of birds scanned of each species amounted to 137,932 Greater White-fronted Geese, 6,695 Greylag Geese, 15,607 Tundra Bean Geese, and 1,780 Barnacle Geese. Inter-specific aggression between species was the very rare event and never shown towards Greylag Geese (Fig. 1B).

### Effects of species, date and goose density on aggressive interactions

**Fig. 2:**
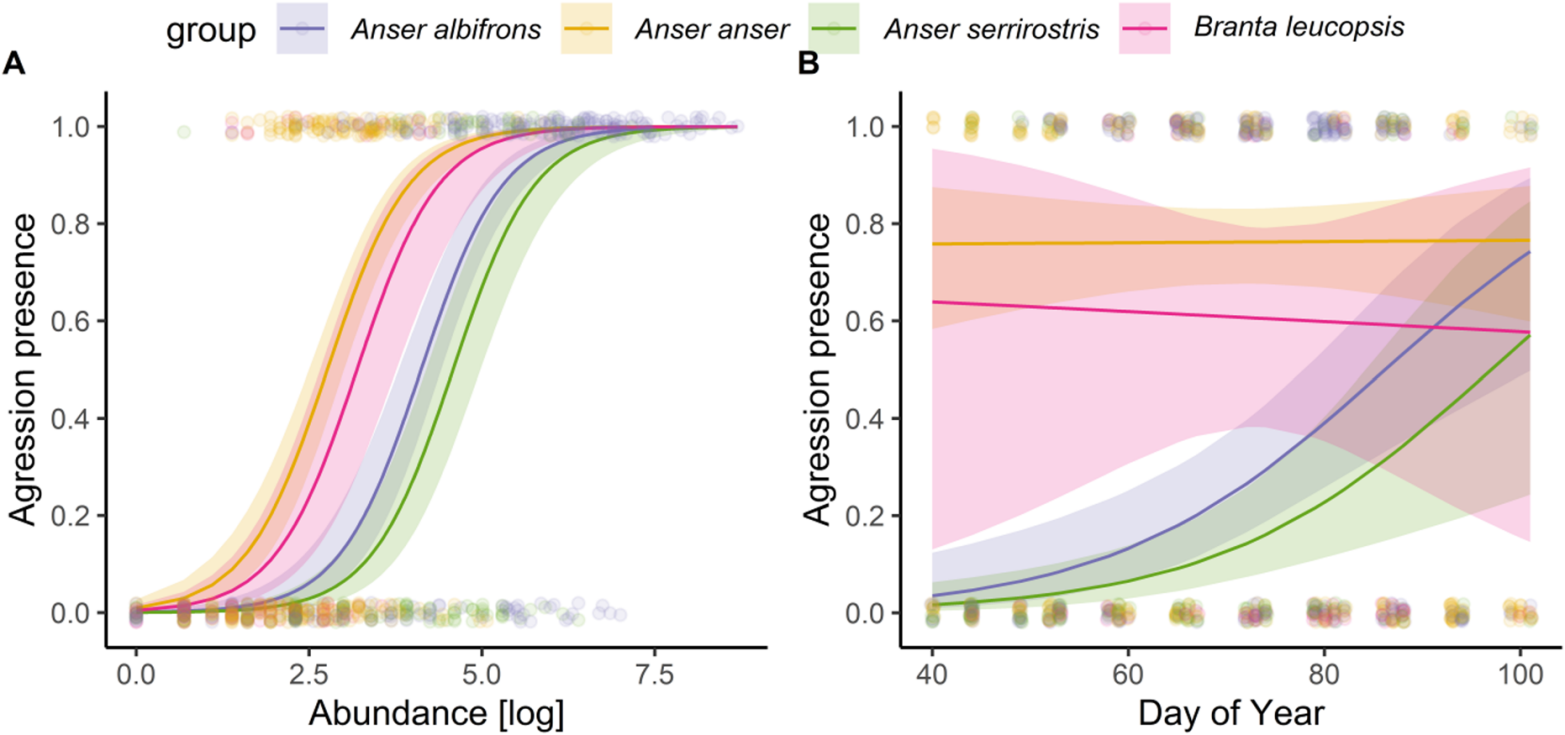
Predicted probability of aggression (per five-minute observation bout) within the four staging goose species (Great White-fronted *Anser albifrons*, Greylag Goose *Anser anser*, Tundra Bean *Anser serrirostris*, Barnacle Goose *Branta leucopsis*) in the Biebrza Basin during spring 2024. Modelled probabilities were generated from the final GLMM as a function of **A** abundance (log-transformed abundance; number of individuals of given species) and **B** day of year. Points represent individual flock observations (jittered horizontally to reduce overlap. The solid lines show species‐specific fitted values from the model on the probability scale, and shaded ribbons denote approximate 95% confidence intervals.

Stepwise removal of non-significant terms (using likelihood-ratio tests at *p* > 0.05) indicated that hour, habitat, and the species × hour as well as species × abundance interactions did not significantly improve model fit; hence, these terms were excluded from the final model. The final model retained species, day of year (doy), abundance, and the species × doy interaction (Table 1). Overall, aggression across all species showed a highly significant (*p* < 0.001) association with abundance (Fig. 2A). There were marked differences in changes in levels of aggression among species. Greylag and Barnacle Geese maintained consistently high overall levels of aggression compared to Greater White-fronted (the reference species in the model was the most abundant and dominant within the study area^27^ and Tundra Bean Geese which did not differ in intercepts (Fig. 2B). In contrast to Greylag and Barnacle Geese, Greater White-fronted and Tundra Bean Geese both showed significant increases in aggression as the season progressed, especially towards the end of the staging period when they approached the levels shown by the other two species (Fig. 2B). There was no effect of habitat (i.e. no difference between cereals fields and grassland, *p* > 0.05).

**Table 1:** Results of the generalized linear mixed model (binomial family, logit link) examining the effects of different factors on the probability of recording aggression per five-minute observation bout in four goose species in the Biebrza Basin during spring 2024.

Fixed Effect	Estimate	Std. Error	z-value	*p*-value
Intercept	− 12.02	1.676	− 7.168	**< 0.001** **
Spec: Greylag	7.302	1.519	4.806	**< 0.001** **
Spec: Bean	− 0.786	1.664	− 0.473	0.64
Spec: Barnacle	6.930	2.854	2.428	0.015 *
Doy	0.072	0.018	4.053	< 0.001 **
Abundance	1.687	0.166	10.158	< 0.001 **
Spec: Greylag × DOY	− 0.071	0.020	− 3.610	< 0.001 **
Spec: Bean × DOY	0.00011	0.023	0.005	0.99
Spec: Barnacle × DOY	− 0.076	0.038	− 2.028	0.043 *

Spec – Species: Greylag (Greylag Goose *Anser anser*), Bean (Tundra Bean *Anser serrirostris*), Barnacle (Barnacle Goose *Branta leucopsis*) and the reference level is White-fronted Goose (*Anser albifrons*); DOY – Day of Year, Abundance – log-transformed abundance. Significant values bolded and marked at *p* < 0.05 = *, *p* < 0.01 = **. Flock identity (flock_id) was included as a random intercept.

## Discussion

These results showed that agonistic interactions in mixed-species goose flocks during spring migration in Poland were almost exclusively intraspecific rather than interspecific. This pattern suggests that the aggressive behaviour observed at staging areas is more consistent with social and reproductive dynamics than with interspecific competition for food, in line with previous work showing that aggression in geese can track temporal proximity to reproduction and investment in pair-bond maintenance^33,34^. Pair bond maintenance and mate guarding in migratory waterbirds are fundamental determinants of reproductive success for both partners in a pair^35^. By remaining close to their partners, and showing aggression towards other flock members, males actively displace rivals, securing uninterrupted opportunities for their associated mate to feed and reducing risks of extra-pair copulations. The nearer to the date of first egg date on the breeding grounds, the more the imperative on the male to indulge in such protective behaviour, hence the date specific function relating to the increase in aggression observed. Although we are obviously unable to distinguish them in the field from interactions resulting from foraging-ground defense, such behaviours become more frequent in larger flocks, where the increased density of potential competitors intensifies social and reproductive interactions^36,37^ confirmed in our observations across all species presented here.

We acknowledge that the scan-sampling methodology limited our ability to directly classify aggressive acts due to the difficulty of reliably determining the sex of individuals in the field and the often challenging assessment of age, which restricts the potential for detailed species-level comparisons. The ephemeral nature of the observed behaviours further complicates the unambiguous assignment of aggressive acts to discrete functional categories, such as mate-guarding versus foraging-ground defence. Goose aggression can serve multiple social functions, including communication, maintaining space, and assessment of conspecifics, and individual variation likely contributes to differences in both the intensity and type of agonistic interactions observed. These methodological and contextual constraints should therefore be considered in designing future research.

Despite these complexities, the temporal patterns of aggression we observed appear to closely track reproductive dynamics, reflecting social and reproductive factors rather than other types of behaviour. Intraspecific aggression increased as females approached the onset of egg-laying, coinciding with intensified foraging activity to accumulate energy reserves necessary for egg production and subsequent self-maintenance during incubation^38^. This heightened female foraging effort was matched by increased mate-guarding activity by males defending females against other males which try to extra-pair copulation. It results in elevated levels of intraspecific aggression nearer the first egg-laying. The absence of any detectable effect of foraging habitat quality on aggression further supports the interpretation that reproductive and social factors, rather than competition for food resources, are the primary drivers of these behaviours.

At spring stopover sites, geese often forage in mobile, densely aggregated large flocks and do not defend fixed territories. Mate-guarding aggression is typically intraspecific and involves brief displacements of conspecifics approaching a pair, often followed by a rapid return to the partner. In light of our results, the observed aggression appears to reflect short-range defence of personal space associated with mate-guarding rather than patch or territorial defence. The predominance of intraspecific interactions and the lack of habitat effects further support this interpretation. Furthermore, the increase in aggression as the season progressed and the breeding period approached is consistent with rising reproductive motivation.

Our study showed major differences in aggression levels between the four species present. Greylag and Barnacle Geese both showed similar (60–80% probability of observation) and unchanged frequency of aggression throughout the spring staging period. However, we should be wary of deriving too many inferences about the Barnacle Geese from these data. This species was not present in large numbers which generated only 16 cases of intra-specific aggression, too few to confidently analyse temporal trends from 26 observation dates. In contrast, Greater White-fronted and Tundra Bean Geese both showed low frequency of aggression in the first month of the staging period and this only began to increase in the last three weeks of observation immediately prior to departure, when the levels of probability began to reach those of the other two species. These differences also support the hypothesis that such behaviours are more related to reproductive investment and mate guarding than conflicts over food resources. Dismissing body size and general levels of aggression in the four species as alternative plausible explanatory variables for these patterns (Barnacle and Greylag Geese are the smallest and largest species of this group, while the other two species show major changes in agonistic interactions with date). Taken together, these interspecific differences are difficult to reconcile with a purely food-limitation explanation for the observed seasonal patterns and are more consistent with changes in social and reproductive motivation (including mate guarding) as spring advances, while recognising that multiple mechanisms may overlap.

Rather this favours support for the hypothesis that the levels of aggression relate to mate guarding across all the species for the following reasons. Wild geese form long-term, often lifetime, pair bonds and maintain close spatial proximity and affiliative behaviours across seasons, including on wintering grounds; this year-round association provides benefits (e.g. higher reproductive success, cooperative vigilance, and facilitation of foraging)^34^. Observational studies on multiple goose species report pairing and mate-maintenance often occur on wintering/staging areas, but there is elevated male vigilance and closer mate-guarding behaviour during the pre-nesting and spring staging period — interpreted as increased mate-guarding (as well as predator-surveillance trade-offs) as females become receptive and paternity/breeding opportunities rise^39,40^. We contend that the predominantly intra-specific nature of the aggression supports the mate-guarding hypothesis, but the nature of the time related patterns among the four species add further weight to this support. Greylag Geese usually lay first eggs in Poland in the second half of March^41^. Hence, this early breeding species rapidly approaching its dates of first egg-laying and therefore it is not surprising that they show consistently high levels of aggression throughout the spring staging period. In contrast, the Greater White-fronted and Tundra Bean Geese continue to the Russian Arctic where their first egg dates are typically in very late May to early June^42^. It is therefore to be expected that while the imperative to mate guard remains high for locally nesting Greylag Geese throughout the spring staging period, for the two Arctic nesting *Anser* species, it makes sense that this behaviour, expressed as increasing frequency of aggression, should only begin to increase in frequency towards the end of the staging period in Poland, as female geese become increasingly receptive to copulation en route to ultimate breeding areas^43^. Unfortunately, the few sampled numbers of Barnacle Geese at the study site provided too little data on aggression frequency to support firm conclusions on this species.

## Conclusions

The predominance of intraspecific interactions, the positive relationship with flock size, and the lack of differences between energetically richer arable lands and energy-poorer grasslands clearly indicate that aggression during spring staging is primarily driven by mate guarding rather than by other motivations, such as competition for food resources. However, methodological limitations prevent us from entirely ruling out alternative drivers of this behavior, which may represent a fruitful direction for future research on these interactions.

## Supplementary Information

Below is the link to the electronic supplementary material.

Supplementary Material 1

Supplementary Material 2

## Data Availability

Data are provided as a supplementary file.
