# Supplementary material for: Intraspecific interactions in spring-staging geese reflect mate guarding and proximity to nesting dates
Source: Sci Rep. 2026 Mar 16;16:13608. doi: 10.1038/s41598-026-43082-x (PMC13121457; doi:10.1038/s41598-026-43082-x)
Supplement: Supplementary file 2 — Supplementary Material 2 [file 41598_2026_43082_MOESM2_ESM.docx]

Title: Intraspecific interactions in spring-staging geese reflect mate guarding and proximity to nesting dates

Running title: Intraspecific interactions of geese during spring stopover

Authors: Michał Polakowski^a^*, Łukasz Jankowiak^a^, Anthony David Fox^b^

^a^ Department of Ecology and Anthropology, Institute of Biology, University of Szczecin, Wąska 13, PL-71-412 Szczecin, Poland

^b^ Department of Ecoscience, Aarhus University, C.F. Møllers Allé 8, DK-8000, Aarhus C, Denmark

*Corresponding author: Michał Polakowski; e-mail: michal.polakowski@usz.edu.pl

ORCID

Michał Polakowski<https://orcid.org/0000-0002-0150-7431>
Łukasz Jankowiak<https://orcid.org/0000-0002-3843-9778>
Anthony D. Fox<https://orcid.org/0000-0001-8083-7633>
